# Navigating One Health in research-for-development: Reflections on the design and implementation of the CGIAR Initiative on One Health

**DOI:** 10.1016/j.onehlt.2024.100710

**Published:** 2024-03-13

**Authors:** Steven Lam, Vivian Hoffmann, Bernard Bett, Eric M. Fèvre, Arshnee Moodley, Chadag Vishnumurthy Mohan, Javier Meteo-Sagasta, Hung Nguyen-Viet

**Affiliations:** a International Livestock Research Institute, Nairobi, Kenya; b International Food and Policy Research Institute, Washington, United States; c Department of Economics and School of Public Policy and Administration, Carleton University, Ottawa, Canada; d Institute of Infection, Veterinary and Ecological Sciences, University of Liverpool, Neston, United Kingdom; e Department of Veterinary and Animal Sciences, University of Copenhagen, Frederiksberg, Denmark; f WorldFish, Penang, Malaysia; g International Water Management Institute, Colombo, Sri Lanka

**Keywords:** Research-for-development, Multisectoral collaboration, One Health, One CGIAR, Process evaluation, Research reform

## Abstract

Adopting One Health approaches is key for addressing interconnected health challenges. Yet, how to best put One Health into practice in research-for-development initiatives aiming to ‘deliver impacts’ remains unclear. Drawing on the CGIAR Initiative on One Health – a global initiative to address zoonotic diseases, antimicrobial resistance, and food and water safety – we reflect on challenges during program conception and implementation, prompting us to suggest improvements in multisectoral collaboration, coordination, and communication. Our approach involves conducting a researcher-centered process evaluation, comprising individual interviews that are subsequently thematically analyzed and synthesized. The key takeaway is that limited time for planning processes and short program timelines compared to envisioned development impacts may impede research-for-development efforts. Yet, collaborative work can be successful when adequate time and resources are allocated for planning with minimal disruption throughout implementation. Additionally, due to the multifaceted nature of One Health initiatives, it is important to pay attention to co-benefits and trade-offs, where taking action in one aspect may yield advantages and disadvantages in another, aiding to identify sustainable One Health development pathways. Forming close partnerships with national governments and local stakeholders is essential not only to promote sustainability but also to ensure local relevance, enhancing the potential for meaningful impact. Finally, regularly assessing progress toward development goals is critical as development stands as an overarching objective.

## Graphical abstract

**Figure:**
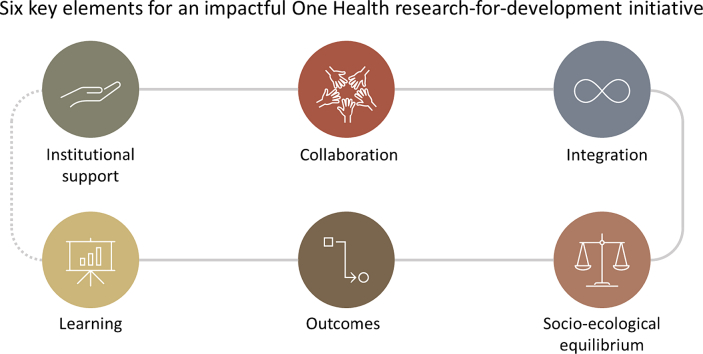

## 1 Background

Collaborative and interdisciplinary approaches, like One Health, are key to addressing interconnected global health threats. These challenges invariably require the involvement of multiple disciplines and sectors. However, putting One Health into practice remains a challenge, as reflected by continued efforts to identify guiding principles [1,2] and develop approaches [3,4], even 20 years after the term ‘One Health’ was first coined [5]. Many factors affecting One Health implementation have been identified, which we have summarized in **Supplementary file: Box S1**. Two main practice and research gaps persist: 1) examples of how to best work together, and 2) evidence on the value-added of using a One Health lens.

Furthermore, One Health implementation is uneven across geographies and focal areas [6]. The understanding of collaborative One Health strategies, particularly in low- and middle-income countries (LMICs), remains relatively under-explored [7,8]. In addition, current attention is focused on coordinated response contexts [[8], [9], [10], [11]] with little reflection on other One Health actions such as research. Researchers engaged in research-for-development initiatives – where research is used as a tool to address development challenges – have insightful experiences working not only across disciplines to generate evidence but also across sectors with partners engaged in practice and policy to foster an environment for evidence use, which are under-documented [12].

CGIAR is a global research partnership dedicated to transforming food, land, and water systems in a climate crisis. In 2021, CGIAR started a research reform by developing a portfolio of 32 research-for-development initiatives. Researchers from CGIAR centres and three external partners (including a donor) came together to develop a proposal for a new initiative on One Health, one of these 32 initiatives. Considering the need for critical reflections on the design and implementation of One Health efforts, the management group of this initiative openly engaged in dialogue addressing the challenges encountered during the first half of the program cycle. We drew on a voluntary process evaluation approach within our team, including interviews with the leads and co‑leads (co-authors) and a synthesis of findings by the evaluation lead (lead author) (for more details on the approach used, see **Supplementary file: Box S2)**. In doing so, this piece will support learning and adaptive management moving into the latter half of the program. Additionally, we hope this piece may improve the success of future research-for-development efforts wanting to use a One Health approach.

## 2 About the CGIAR Initiative on One Health

**Fig. 1:**
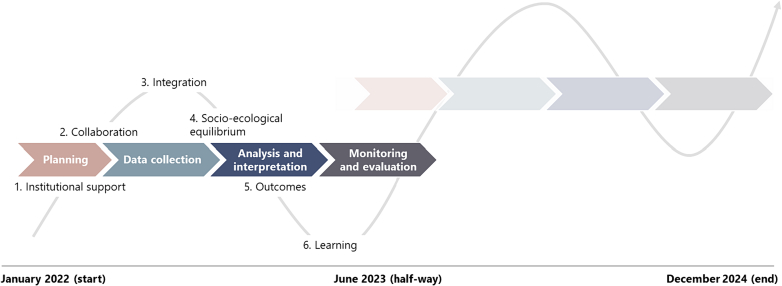
Reflections on One Health collaborative elements and their relation to different phases of a project cycle.

The CGIAR Initiative on One Health aims to address zoonotic and foodborne diseases and antimicrobial resistance in food systems in LMIC settings. The initiative is operationalized through five thematic work packages (WPs) **(Supplementary file: Box S3).** Each WP aims to generate contextually relevant evidence and solutions, develop capacity for their use, and engage in policy and practice change (**Supplementary file**: **Fig. S1**). WPs target change at the community, national, and/or international levels in seven countries: Vietnam, India, Bangladesh, Ethiopia, Kenya, Uganda, and Ivory Coast **(Supplementary file: Fig. S2**). The proposed outcomes vary by WP, reflecting different starting conditions. For instance, in Vietnam and Ethiopia, we are helping to set up technical working groups within pre-existing One Health frameworks. However, due to variations in the timing of these structures' establishment, differing levels of effort are necessary. Below, we share six reflections, which correspond to different general phases of a project cycle (Fig. 1).

## 3 Reflection 1: Institutional support

Within our group, we had strong support from the senior leadership team who helped to bring together experts from different CGIAR centres, embed One Health principles in key documents, and build awareness of its importance across institutional and partner websites. Additionally, external reviewers offered valuable feedback and direction on our work. We had freedom to design projects that expanded on existing work from the CGIAR research programs (CRPs) – the previous research program of the CGIAR (2012 to 2021) – particularly the CRP on Agriculture for Nutrition and Health (A4NH). This continuity supports a move from short-term projects to longer-term research programs, which is key for building momentum and long-term partnerships [13].

Several institutional barriers however made developing and executing an impactful initiative difficult. Firstly, the accelerated proposal development process limited the opportunities for in-depth discussions, identification and engagement of key members, reflections, and planning for cross-WP activities, which hindered extensive cross-WP collaborations. Critical aspects – such as sustainability and scalability – might also not have received the necessary attention. Secondly, delays in funding confirmation – and eventual funding cuts – necessitated last-minute adjustments, resulting in, for example, scaling-down activities. This in turn posed challenges in sustaining relationships with our implementing partners. Finally, the 3-year program cycle is short to have the desired development outcomes we had set out to achieve. The disconnect between the time and resources needed to achieve outcomes and the lifespan of research activities echoes the experiences of CRPs [14].

While institutional challenges are not uncommon in collaborative research projects, we consider these barriers as interconnected with additional challenges discussed below. There are complexities in developing a unified One Health research program, which hinges on robust stakeholder and country consultations in the conception period. Institutions could catalyze such well-rooted research if additional time and resources were allocated to ensure all the required elements are thoughtfully considered during program conceptualization. The initial six-month proposal writing period might have sufficed, but with additional proposal elements requested at a later time, it would have been helpful to extend the timeline or clearly outline all required information from the outset. Funders should consider funding proposals explicitly and view the proposals themselves as valuable outputs.

## 4 Reflection 2: Collaboration

CGIAR collaborates with nearly 2000 active partners spanning 126 countries, including national agricultural research and extension systems, United Nations agencies, universities, research institutions, civil society organizations, and enterprises of various sizes [15]. All CGIAR centres have a history of collaboration in the area of agriculture for nutrition and health, and more recently, in response to the COVID-19 pandemic, many have further united under the One Health framework [16]. However, this move toward a more holistic, integrated approach presented challenges. There were assumptions and misunderstandings about not only institutional working practices but also expectations of the roles to be played by various team members. At the start, taking the time to understand how different centres function, defining explicit expectations of roles within joint projects, and making joint decisions about how to appropriately staff and fund collaborative projects would have facilitated a smoother team collaboration and stronger, more harmonized implementation.

The literature stresses the need to make time to work together, develop communication methods, and establish collaborative mechanisms for success in One Health efforts [7,17]. It is easy to lose sight of these strategies in research-for-development programs with many research and non-research partners. This is especially true for institutions facing funding uncertainty, which may, as a risk mitigation strategy, pursue efforts that have less scope and fewer partners. A key lesson here is the importance of keeping a consistent budget and timeframe for collaborations encompassing diverse partners, from research and implementation to policy.

Our initiative was developed by CGIAR centres that generally focus on animal and aquatic food systems and their links to the environment, food safety, and human health. Collaborations with external partners, such as universities and research institutes, with expertise in public health, wildlife, and disease ecology helped to broaden the range of perspectives, enriching the initiative's design. However, we acknowledge the need for sustained engagement, as the initial strong involvement had declined during the implementation phase, largely due to insufficient funding to include them as project partners. Additionally, during the proposal development phase, while seven consultations were conducted at both country and regional levels, pinpointing priority areas for proposal development, engagement with national partners also diminished during implementation. Partnerships with local partners and national governments are key not only for the scalability and sustainability of these initiatives but also for fostering local relevance, which in turn enhances the potential for meaningful impact.

## 5 Reflection 3: Integration

We had discussions about integration, commonly viewed as weaving together concepts and methods from different disciplines to develop a more holistic understanding of a problem and find a more complete solution [18]. This approach to integration was agreed upon in the proposal. The arrangement of WPs by thematic areas allowed us to first focus on solid disciplinary work, which is considered a prerequisite for effective integration in One Health contexts [19]. However, because WPs independently addressed different facets of One Health within their respective thematic areas, in turn, there was a lack of opportunities to synergize, pool resources, work on common value chains, and address broader questions, potentially leading to the untapped One Health potential of the initiative.

It is important to note that One Health integration can take various forms [9]. Generally, integration worked well where WPs shared commonalities in methodologies, country activities, workspaces, and capitalization of shared resources. For example, cross-WP collaborations in Ethiopia and Vietnam will measure the impact of participation in food safety training on both economic and microbiological outcomes (WP2 and WP5). WP3 and WP4 are monitoring pathogens and resistomes using similar methods but in different ecological niches (e.g. in livestock and aquaculture farms for WP3 and watersheds for WP4). Often One Health issues are addressed in isolation [10]; the integration we achieved between WPs might not have been realized had we not given priority to fostering integration among them.

## 6 Reflection 4: Socio-ecological equilibrium

A core principle of One Health is to achieve a harmonious balance between human-animal-environment interaction [20]. The CGIAR One Health Initiative was designed to target the improvement of human health as the ultimate outcome, with the understanding that healthy animals and environment are required to achieve this goal. While organizing the research agenda around a single outcome facilitated prioritization, we acknowledge more could be done to consider the animal and environmental health dimensions of One Health.

One Health efforts also operate in complex and dynamic systems. Systems thinking is a promising strategy for considering the other dimensions of One Health by making explicit connections between the different WPs, as well as WPs and the agri-food systems in which WPs are introduced [21]. Furthermore, because optimizing the health of humans, animals, and the environment, simultaneously, is difficult, being cognizant of trade-offs could help ensure research with the highest net benefits and lowest risk of negative impacts is conducted. Paying attention to co-benefits – where taking action in one dimension provides advantages to another – also serves to identify sustainable One Health development pathways [22]. Finally, expanding community outreach efforts, such as establishing One Health field sites [23], could enhance awareness and actions concerning health risks at the socio-ecological interface.

## 7 Reflection 5: Outcomes

Tracking outcomes is important for understanding what we are doing well and where we fall short in terms of collaboration. We have successfully undertaken strong applied research, laying the groundwork for several large-scale studies that will begin in the second half of programming, including for example: established a strong working relationship with government authorities in Kajiado, Kenya, who have officially committed funding from government budgets for personnel to work with us on a zoonoses surveillance program; designed protocols for randomized controlled trials testing the impact of food safety training on business outcomes and food safety among small-scale meat vendors in Vietnam and Ethiopia; conducted observational studies on the use of antimicrobials in poultry (Kenya) and fish (Bangladesh) production to inform strategies to tackle antimicrobial resistance; characterized health and nutritional impact of bush meat consumption in Côte d'Ivoire, and developed pollution monitoring plans for watersheds in Ethiopia and India [24].

Our research-for-development initiative ultimately seeks to bring about positive change. As much of our work builds on the already strong foundation with long-standing partners, it is imperative to transition from research to the *use of research*. At this stage, halfway through the Initiative, we are pursuing strategies to move research along the impact pathway as we are doing the science/evidence generation. Up to this point, the team has engaged in various international advisory groups of the Quadripartite and disseminated results at national, regional, and international levels. Moving forward, our future activities will include developing external communication products based on joint activities and crafting unified messages across thematic areas. Some WPs have processes for engaging governmental actors, such as sharing evidence summaries every six months, which is being considered by other WPs. Other WPs are developing stakeholder engagement plans to identify relevant One Health stakeholders and mechanisms for engagement, particularly with national partners who could help support scalability and sustainability. When there is political will to addressing One Health challenges and promoting evidence-informed decision-making, research findings are more likely to be incorporated into policies and programs, which could result in more effective and widespread outcomes.

## 8 Reflection 6: Learning

In a systematic review of challenges in conducting One Health initiatives, the biggest knowledge gap was the lack of monitoring and evaluation studies demonstrating what works and why [8], highlighting the need for learning from One Health efforts. Qualitative evaluations are particularly absent [10]. This piece is a product of a reflection exercise conducted to qualitatively evaluate our initiative's processes. Furthermore, toward the end of year 1, we held our first virtual ‘pause-and-reflect’ session to share progress and identify entry points for integration. At our annual planning meeting held in April 2023, we also conducted in-person reflective sessions. Subsequently, we have hosted several meetings focused on complementary work, with additional ones scheduled for the future. However, acknowledging the cost and environmental implications of in-person meetings, some of these will continue to be conducted virtually. Consistent communication with the CGIAR initiative management unit via monthly 2-h management meetings, coupled with the adoption of a shared results framework across various initiatives, helped us document progress toward our outcomes.

We are using Theory of Change and other evaluation frameworks to support us in learning about our initiative's outcomes [25,26]. Efforts to define and assess outcomes are generally categorized into three main areas: (a) outputs, or indicators of proper functioning of the collaborative itself; (b) intermediate results, or tangible changes resulting from the collaborative's actions; and (c) impact, or the long-term changes brought about by these actions. Given our programs' level of maturity and the time lags between development actions and outcomes, we have chosen to prioritize the assessment of the intermediate results/outcomes level. Part of this process involves reviewing our outputs and examining the potential results they may lead to. At the halfway mark of our program, spanning 1.5 years, we have achieved commendable outputs within our budget allocation, including various knowledge products, capacity-sharing initiatives for development, and innovation developments – all meticulously monitored via a dashboard [27] – which could lead to outcomes. We are finding this approach is well-suited for tracking and learning from the gradual progress that has been achieved in our research-for-development efforts. It is worth noting that having an evaluation specialist on the team greatly supports this learning process.

## 9 Implications for research and practice

In line with reviews highlighting the factors that influence the success of One Health initiatives [7,8], we echo the importance of conditions for starting, existing structures, and organizational factors, which we have categorized as institutional supports. Our contribution to the literature lies in providing a more nuanced perspective on One Health collaborations, particularly within the realm of research-for-development contexts, where the objectives encompass both research and development. This requires innovative approaches to planning and evaluation.

Due to their multifaceted nature, research-for-development efforts require collaboration across various stakeholders, fostering environments where downstream outcomes can manifest [12]. It is the productive interactions among participants, rather than the individual actions of actors, that are key to creating the conditions for outcomes to emerge. During the planning phase, it may thus be important to allocate more time and resources for research-for-development efforts (or maintain the same time and resources with minimal changes), compared to initiatives exclusively centred on either research or development, to ensure careful considerations of collaboration dynamics.

In terms of evaluation, solely examining downstream results will fail to capture the important underlying processes and conditions, making it essential to design evaluation questions that delve into the ‘how’ and ‘why’ of outcomes, thus offering insights into the relational mechanisms driving outcomes. Reviews of One Health processes and outcomes have primarily focused on quantitative indicators [10,28,29], overlooking the role of qualitative evaluations. As this study and others show [9], qualitative assessments can not only provide deeper insights into effective collaboration mechanisms but also shed light on the factors contributing to the success, as well as their appropriateness in diverse contexts, which can be helpful when scaling up efforts.

## 10 Conclusion

This piece presents six early reflections from implementing a One Health research-for-development initiative, operationalized through five WPs with activities in seven countries (Box 1). Despite the variability in WPs and country settings, the experiences of the initiative revealed some commonalities, resulting in practical considerations.

**Box 1:**
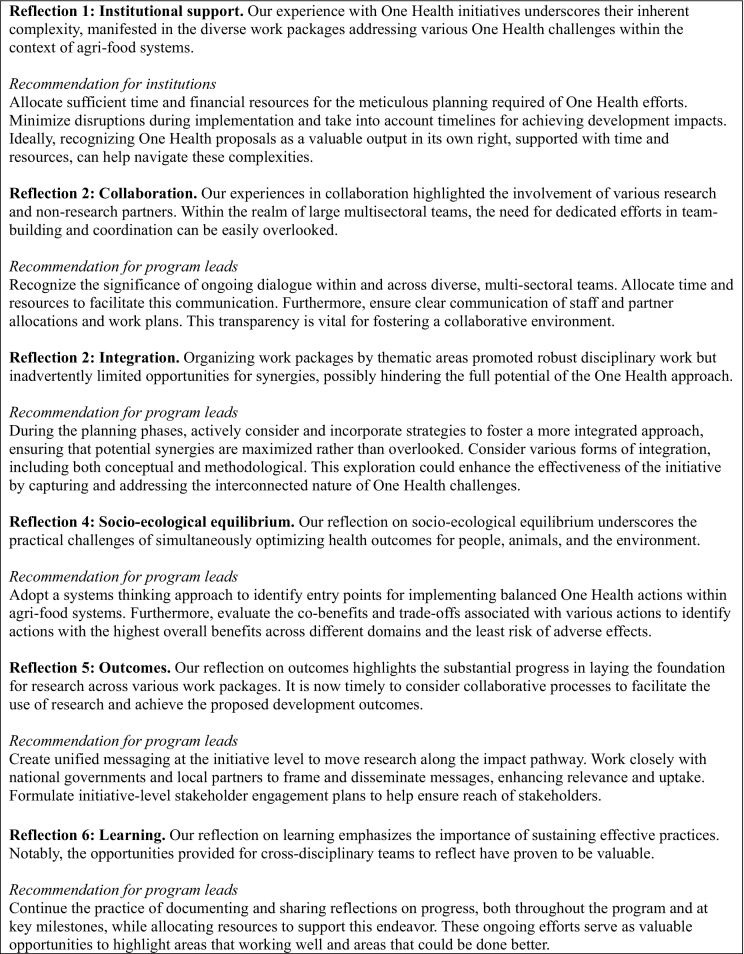
Reflections and recommendations for an impactful One Health research-for-development initiative

Firstly, it is imperative to allocate sufficient time and resources, especially during the planning phase, to comprehensively address the essential components of multifaceted endeavors. Secondly, it is critical to foster strong partnerships with national governments and local partners, which are vital not only for the scalability and sustainability of these initiatives but also for fostering local relevance, which in turn enhances the potential for meaningful impact. Thirdly, One Health initiatives demand a nuanced consideration of trade-offs and co-benefits; this involves identifying actions that offer the greatest benefits across different domains while minimizing the risk of adverse effects. Lastly, owing to its overarching developmental objectives, it becomes critical to deliberate on the next steps, particularly around the use of research findings; regularly assessing progress toward the attainment of development goals, and not only research goals, plays a key role in this regard.

We note several limitations of our approach. Firstly, our self-reflections centered on our personal experiences as CGIAR researchers engaged in the One Health collaboration, potentially neglecting broader perspectives. While the planning and implementation of our initiative primarily involved CGIAR centers, engaging with our broader research team and partners could have corroborated our findings and provided additional insights. Secondly, although we acknowledge the complexity and dynamism inherent in One Health efforts, and the potential benefits of employing a systems thinking approach for program development and evaluation, we did not use such an approach in this process evaluation. This decision was driven by our primary focus on actionable ideas to support implementation in the latter half of the program. Future evaluations could benefit from adopting a systems thinking or another similar lens (e.g. Theory of Change) by placing attention on not only the different dimensions of One Health but also cross-cutting themes such as climate change and gender and social equity [30,31]. Nonetheless, this work offers key insights into process from researchers involved in diverse One Health topics.

We trust these experiences of the CGIAR One Health initiative can provide researchers and development practitioners with a strong foundation for One Health research-for-development, enabling them to plan and execute collaborative work more effectively. This is particularly important in the context of the emerging infectious disease outbreaks and the need for increased adoption of One Health to prevent the next pandemic and other global health challenges.

**Table:** 


**Reflection 1: Institutional support.** Our experience with One Health initiatives underscores their inherent complexity, manifested in the diverse work packages addressing various One Health challenges within the context of agri-food systems. *Recommendation for institutions* Allocate sufficient time and financial resources for the meticulous planning required of One Health efforts. Minimize disruptions during implementation and take into account timelines for achieving development impacts. Ideally, recognizing One Health proposals as a valuable output in its own right, supported with time and resources, can help navigate these complexities. **Reflection 2: Collaboration.** Our experiences in collaboration highlighted the involvement of various research and non-research partners. Within the realm of large multisectoral teams, the need for dedicated efforts in team-building and coordination can be easily overlooked. *Recommendation for program leads* Recognize the significance of ongoing dialogue within and across diverse, multi-sectoral teams. Allocate time and resources to facilitate this communication. Furthermore, ensure clear communication of staff and partner allocations and work plans. This transparency is vital for fostering a collaborative environment. **Reflection 2: Integration.** Organizing work packages by thematic areas promoted robust disciplinary work but inadvertently limited opportunities for synergies, possibly hindering the full potential of the One Health approach. *Recommendation for program leads* During the planning phases, actively consider and incorporate strategies to foster a more integrated approach, ensuring that potential synergies are maximized rather than overlooked. Consider various forms of integration, including both conceptual and methodological. This exploration could enhance the effectiveness of the initiative by capturing and addressing the interconnected nature of One Health challenges. **Reflection 4: Socio-ecological equilibrium.** Our reflection on socio-ecological equilibrium underscores the practical challenges of simultaneously optimizing health outcomes for people, animals, and the environment. *Recommendation for program leads* Adopt a systems thinking approach to identify entry points for implementing balanced One Health actions within agri-food systems. Furthermore, evaluate the co-benefits and trade-offs associated with various actions to identify actions with the highest overall benefits across different domains and the least risk of adverse effects. **Reflection 5: Outcomes.** Our reflection on outcomes highlights the substantial progress in laying the foundation for research across various work packages. It is now timely to consider collaborative processes to facilitate the use of research and achieve the proposed development outcomes. *Recommendation for program leads* Create unified messaging at the initiative level to move research along the impact pathway. Work closely with national governments and local partners to frame and disseminate messages, enhancing relevance and uptake. Formulate initiative-level stakeholder engagement plans to help ensure reach of stakeholders. **Reflection 6: Learning.** Our reflection on learning emphasizes the importance of sustaining effective practices. Notably, the opportunities provided for cross-disciplinary teams to reflect have proven to be valuable. *Recommendation for program leads* Continue the practice of documenting and sharing reflections on progress, both throughout the program and at key milestones, while allocating resources to support this endeavor. These ongoing efforts serve as valuable opportunities to highlight areas that working well and areas that could be done better.

Alt-text: Unlabelled Box

## CRediT authorship contribution statement

**Steven Lam:** Conceptualization, Data curation, Formal analysis, Methodology, Writing – original draft, Writing – review & editing. **Vivian Hoffmann:** Conceptualization, Supervision, Writing – review & editing. **Bernard Bett:** Writing – review & editing. **Eric M. Fèvre:** Writing – review & editing. **Arshnee Moodley:** Writing – review & editing. **Chadag Vishnumurthy Mohan:** Conceptualization, Writing – review & editing. **Javier Meteo-Sagasta:** Conceptualization, Writing – review & editing. **Hung Nguyen-Viet:** Conceptualization, Supervision, Writing – review & editing.

## Declaration of competing interest

No conflict of interest is stated.

## Data Availability

No data was used for the research described in the article.

## Appendix A Supplementary data

Supplementary material Image 1
