# Supplementary material for: Navigating One Health in research-for-development: Reflections on the design and implementation of the CGIAR Initiative on One Health
Source: One Health. 2024 Mar 13;18:100710. doi: 10.1016/j.onehlt.2024.100710 (PMC10963858; doi:10.1016/j.onehlt.2024.100710)
Supplement: Supplementary file 1 — Supplementary material [file mmc1.docx]

**Supplementary file**

| **Box S1.** Factors affecting One Health research |
| --- |
| A scoping review of multisectoral collaborations focused on infectious disease identified factors that support successful One Health collaborations, grouped across three levels [1]:   1. **Individual factors** (education & training and prior experience & existing relationships) 2. **Organizational factors** (organizational structures, culture, human resources, and communication) 3. **Network factors** (networks structures, relationships, leadership, management, available & accessible resources, and political environment)   A systematic review of One Health initiatives generally identified the following challenges, grouped in three distinct chronological phases [2]:   1. **Conditions for starting** (policy & funding, education & training) 2. **Execution** (surveillance, multi-actor collaborations, multi-domain collaborations, multi-level collaborations) 3. **Monitoring and evaluation** (evidence)   A scoping review of evaluations of One Health initiatives targeting infectious diseases and antimicrobial resistance identified the following facilitators to the success of these initiatives [3]:   1. **Structural / economic** (available/existing structures, low cost of intervention) 2. **Social** (socially and culturally appropriate) 3. **Political** (health perceived as a priority) 4. **Communication / coordination** (collaboration and communication between stakeholders) 5. **Methodological** (continually review and revise protocols) 6. **Paradigm / ontology** (One Health thinking) 7. **Gap of awareness, education / training** (recognition and awareness of One Health evidence) |

| **Box S2.** Process evaluation approach |
| --- |
| **Data collection.** We developed a semi-structured interview guide with process evaluation questions related to implementation (e.g. process, challenges, successes), outcomes (including for whom), and contextual factors influencing implementation and outcomes (including climate change). Because equity and climate action are cross-cutting themes of the initiative, we integrated these considerations into the interview guide. Interviews were conducted between May and June September 2023 by the lead author. Interviews were audio-recorded with verbal informed consent and varied from 60 to 90 mins. Interviews were transcribed verbatim for analysis.  **Analysis.** We conducted thematic analysis to identify patterns or themes in the data [4]. Specifically, we applied several sequential phases of deductive coding, with results from one phase informing the focus of coding in the subsequent phase. In phase 1, we examined perceptions on implementation. Building from these findings, phase 2 focused on preliminary outcomes resulting from implementation. Finally, in phase 3, we explored contextual factors influencing implementation and outcomes. The draft themes were first synthesized by the lead author, with regular discussions held among the authorship team to further develop the themes. |

| **Box S3.** CGIAR Initiative on One Health work packages at a glance |
| --- |
| We are contributing to the evidence base that:   - Surveillance of and evidence on zoonotic diseases allows governments – at different levels – better implement large-scale control **(work package 1: zoonoses)** - Randomized controlled trial intervention packages can enhance hygienic practices in small scale slaughterhouses and improve food safety in traditional markets **(work package 2: food safety)** - Reductions in antimicrobial use in poultry, pig, and aquaculture systems do not affect profits **(work package 3: antimicrobial resistance)** - Water quality monitoring and modeling can support watershed decision-making **(work package 4: water safety)** - Consideration of incentives and constraints facilitates the adoption of evidence and innovations **(work package 5: economics, governance, and behaviour)** |

**
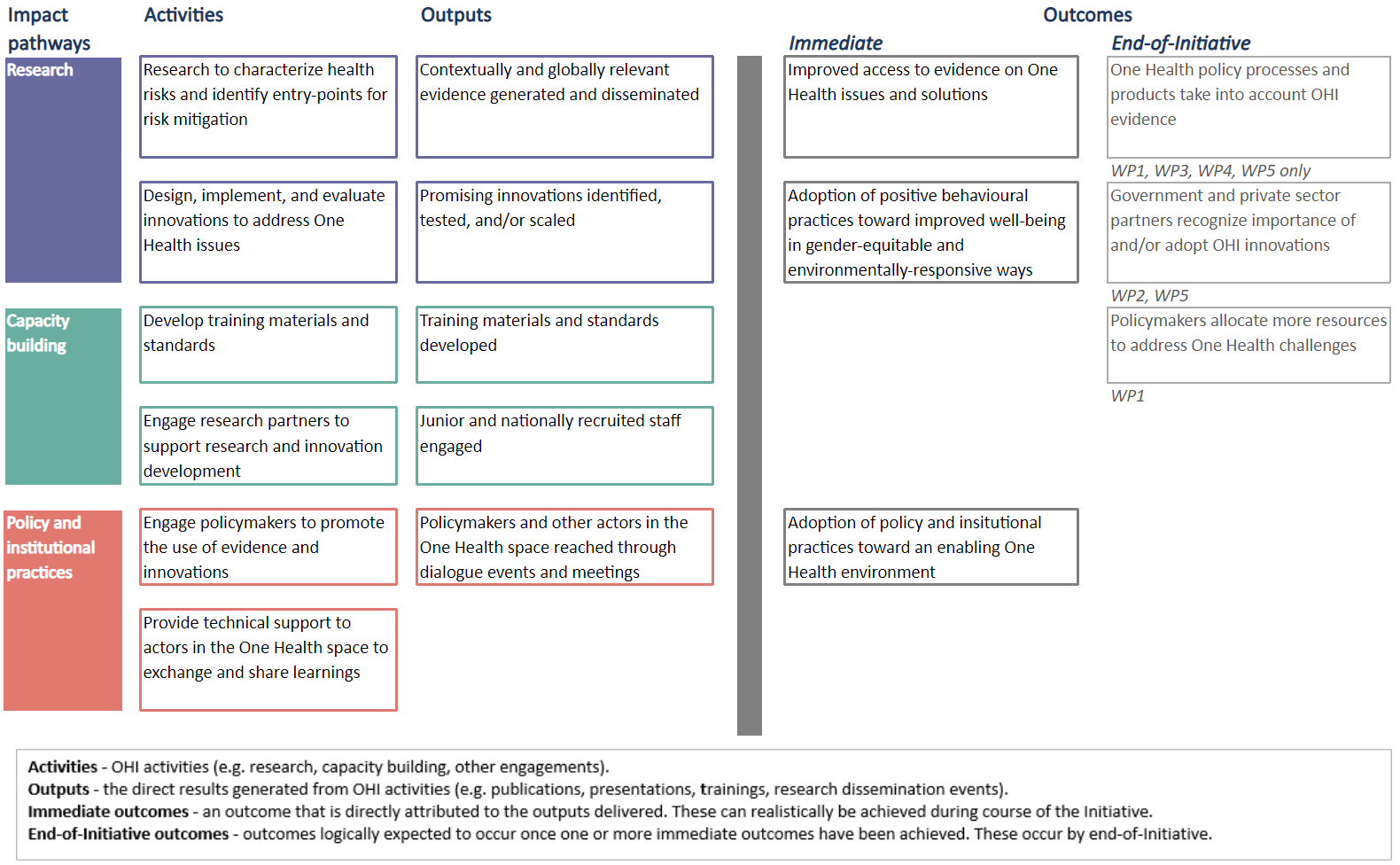
**

**Figure S1.** Theory of Change for the CGIAR Initiative on One Health.


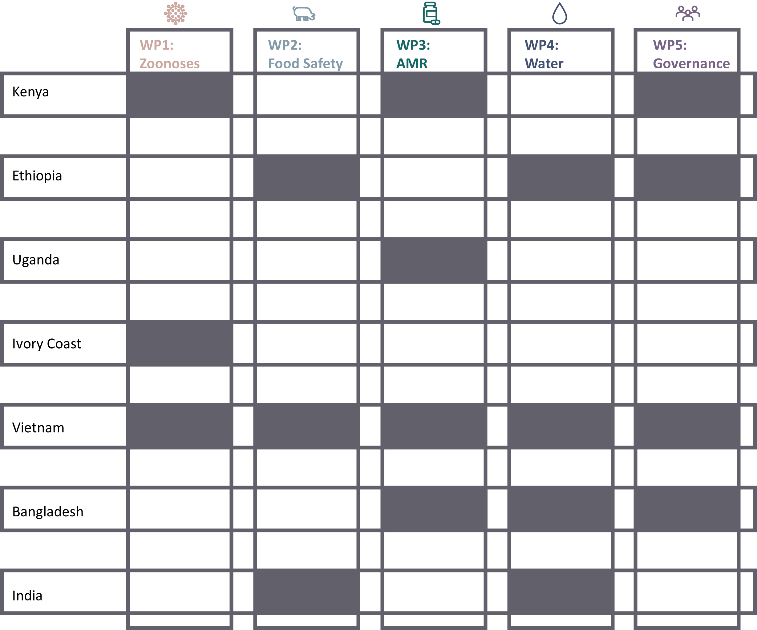


**Figure S2.** CGIAR Initiative on One Health work packages (WPs) and study countries.

**References**

[1] Errecaborde KM, Macy KW, Pekol A, Perez S, O’Brien MK, Allen I, et al. Factors that enable effective One Health collaborations - A scoping review of the literature. PLoS One 2019;14:e0224660. https://doi.org/10.1371/journal.pone.0224660.

[2] dos S. Ribeiro C, van de Burgwal LHM, Regeer BJ. Overcoming challenges for designing and implementing the One Health approach: A systematic review of the literature. One Health 2019;7:100085. https://doi.org/10.1016/j.onehlt.2019.100085.

[3] Delesalle L, Sadoine ML, Mediouni S, Denis-Robichaud J, Zinszer K, Zarowsky C, et al. How are large-scale One Health initiatives targeting infectious diseases and antimicrobial resistance evaluated? A scoping review. One Health 2022;7:1000085. https://doi.org/10.1016/j.onehlt.2022.100380.

[4] Braun V, Clarke V, Hayfield N, Terry G. Thematic analysis. In: Liamputtong P, editor. Handb. Res. Methods Heal. Soc. Sci., Singapore: 2018.
